# An evaluation of alcohol attendances to an inner city emergency department before and after the introduction of the UK Licensing Act 2003

**DOI:** 10.1186/1471-2458-8-379

**Published:** 2008-10-31

**Authors:** AJ Durnford, TJ Perkins, JM Perry

**Affiliations:** 1 School of Medicine, University of Birmingham, Edgbaston, Birmingham, B15 2TT, UK

## Abstract

**Background:**

The Licensing Act 2003 (The Act) was implemented on the 24th November 2005 across England and Wales. The Act allowed more flexible and longer opening hours for licensed premises. We investigated the effect of The Act on alcohol related attendances to an inner city emergency department in Birmingham, UK.

**Methods:**

We compared the proportion and time of alcohol related emergency department attendances in one week periods in January 2005 and 2006, before and after the implementation of The Licensing Act 2003. An alcohol related attendance was defined as any attendance where there was any documentation of the patient having consumed alcohol before presenting to the emergency department, if they appeared intoxicated on examination, or if alcohol attributed to their final diagnosis.

**Results:**

The total weekly attendances increased slightly from 1,912 in 2005 to 2,146 in 2006.

There was non-significant reduction in the proportion of alcohol related attendances between 2005 (3.6%) and 2006 (2.9%). A significantly greater proportion of attendances occurred at the weekend between 18.00 and 23.59 in 2005 (61.4%) than in 2006 (17.2%). There was a corresponding significant increase in the weekend proportion of attendances occurring between 03.00 to 05.59 in 2006.

**Conclusion:**

Our findings show that there was a change in the pattern of alcohol related attendances to the emergency department around the time of implementation of the Licensing Act 2003, which has implications for delivery of emergency department services.

## Background

Alcohol has an acknowledged negative impact on individual health and societies, with 4% of the global burden of disease attributable to its misuse, comparable to the impact of tobacco and hypertension [1]. Alcohol-related deaths rose by one fifth over a recent 5 year period with the burden on the National Health Service (NHS) due to alcohol related problems increasing and the costs to the NHS estimated at £1.7 billion [2-4]. Alcohol is responsible for around 150,000 hospital admissions in England and Wales each year, the majority of which occur between 12.00 and 05.00 [5].

Levels of alcohol consumption are affected by its availability and the occurrence of alcohol-related problems including alcohol related illness and injury rises with availability [6,7]. The availability and consumption of alcohol is determined by many factors, including price and accessibility [8].

The Licensing Act 2003, implemented in England and Wales on the 24^th ^November 2005 [9], allowed more flexible opening hours for licensed premises, with the potential for opening up to 24 hours a day, seven days a week. There were four objectives: i) the prevention of crime and disorder, ii) public safety, iii) the prevention of public nuisance, and iv) the protection of children from harm. The government believed that achieving these objectives would lead to the public in England and Wales adopting a more relaxed approach to drinking, resulting in fewer alcohol related crimes, health issues and violence [10]. Some commentators remained sceptical [11], and early evidence suggests that there has been little change in drinking habits since the introduction of the Act [12]. More recently, one study of overnight attendances to a UK emergency department found a significant rise in alcohol related attendances from 2.9% to 8% since the Act [13]. Another study of alcohol related attendances at an emergency department in the United Kingdom found that restricting the availability of extensions to licensing hours led to no significant changes in the pattern of alcohol related attendance [14].

The aim of our study is to assess the impact of the Licensing Act 2003 upon alcohol related attendances to a busy emergency department of an inner city hospital in Birmingham, England.

## Methods

We analysed the electronic triage summary records of all people attending the emergency department of City Hospital, an inner city hospital in Birmingham, during one week in January 2005 (before the Act) and January 2006 (after the Act) to determine if their presenting complaint was alcohol related. The records were manually scrutinized for any documentation that the patient had been, or appeared to have been, drinking immediately prior to attendance, or admitted being intoxicated due to alcohol upon examination, or if alcohol attributed to their final diagnosis. We excluded patients with alcohol-related medical problems who had no documentation of recent alcohol consumption prior to attendance. Attendances which fulfilled these criteria were defined as alcohol related. Where attendance was alcohol related we recorded the time and date of arrival to the emergency department, the patients age and sex, and whether the patient required admission to hospital.

We chose the last week in January (25^th ^January–1^st ^February 2005; 24^th ^January–31^st^January) to undertake the evaluation so that attendances were not directly affected by events such as Christmas and that monthly-paid employees would have access to similar levels of money to spend. Additionally there were no scheduled large sporting or musical events during this week. We defined a week from 09:00 Tuesday to 08:59 the following Tuesday, with each day defined as a 24 hr period commencing at 09.00, and defined a weekend as from 18.00 on Friday to 08.59 on a Monday morning. We grouped the time of attendance into 3 hr blocks. We analysed our results using the 95% confidence limits around the difference between proportions, Chi-Squared methods or Fishers exact test where appropriate.

To check for misclassification in case ascertainment we took a random sample (using random number tables) of 100 attendances we had classified as non-alcohol related and re-scrutinized the medical records directly for any documentation of alcohol involvement. We found no evidence of misclassification in our random sample of 100 attendances not related to alcohol. In addition we also screened 50 of our positive results and found no cases of misclassification.

## Results

There were 1,912 attendances to the emergency department in the chosen week in January 2005 of which 69 (3.6%) were alcohol related. In 2006 there were 2146 attendances with 62 (2.9%) related to alcohol, a difference that was not statistically significant (0.7%, 95% confidence interval --0.4% to 1.8%; χ^2 ^= 1.676; p = 0.195). The median age of alcohol related attendees was 29.5 years (interquartile range 21–44.5) in 2005 and 32 years (range 20–47) in 2006. In 2005 62.3% of attendees were male, compared to 64.5% in 2006. The number of alcohol related attendances resulting in admission to hospital were not significantly different with 10 admissions (10/69; 14.5%) in 2005 and 7 (7/62 11.3%) in 2006 (p = 0.586).

**Figure 1:**
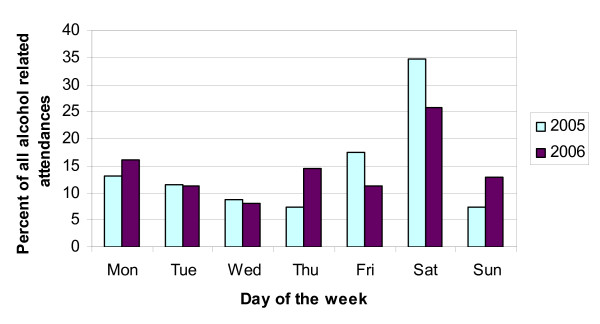
The distribution of alcohol related attendances by day of the week.

The distribution of alcohol related attendances to the emergency department varied according to the day of the week (Figure 1), with the greatest proportion of alcohol related attendances occurring on Saturday in both 2005 and 2006. There were no statistically significant differences between the comparative days of the week in 2005 and 2006.

**Figure 2:**
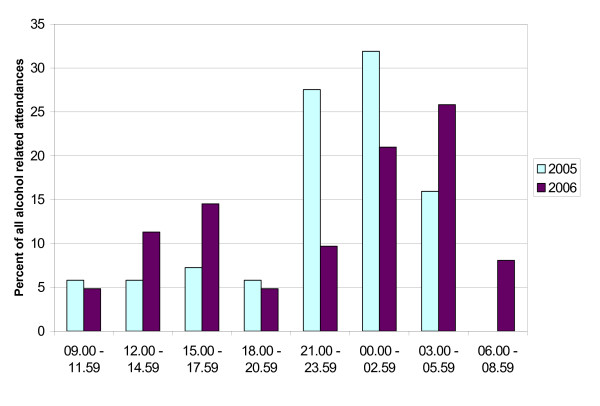
The distribution of alcohol related attendances by time of day.

The distribution of alcohol related attendances varied according to the time of day (Figure 2 and Table 1). There was a significant reduction in the proportion attending between 21.00 and 23.59, with a significant increase in the proportion of attendances between 21.00 and 02.59. A similar proportion of alcohol related attendances occurred after midnight and before 06.00 am (47.8% versus 46.8%) but in 2006 more of these attendances were between 03.00 and 05.59. There was a non-statistically significant greater proportion of alcohol related attendances during normal working hours in 2006 than in 2005 (30.6% versus 18.8%, difference 11.8%, 95% confidence interval 26.5% to -2.9%, p = 0.117).

**Table 1:** Proportion of alcohol related attendances by time of day at weekends and weekdays

	**2005 %**	**2006 %**	**Difference**	**95% confidence interval**		**P value**
				**Lower limit**	**Upper limit**	

**Total attendances**	3.6	2.9	0.7	-0.4	1.8	0.195

**12.00 – 14.59**	0.6	1.1	0.5	-15.1	4.2	0.299

**15.00 – 17.59**	0.7	1.5	0.7	-18.0	3.4	0.228

**18.00 – 20.59**	0.6	0.5	0.1	-6.7	8.7	0.817

**21.00 – 23.59**	27.5	10.0	17.5	4.9	30.7	0.031

**00.00 – 02.59**	31.9	21.0	10.9	-4.1	25.9	0.282

**03.00 – 05.59**	15.9	25.8	9.9	-23.8	4.0	0.259

**06.00 – 08.59**	0	0.8	0.8	-14.9	-1.30	0.021

**Weekend**	6.1	3.9	2.2	-0.1	4.6	0.069

**12.00 – 14.59**	0.0	6.9	6.9	-16.1	2.3	0.178

**15.00 – 17.59**	2.6	13.8	11.2	-24.7	2.3	0.155

**18.00 – 20.59**	25.6	10.3	15.3	-2.4	32.9	0.113

**21.00 – 23.59**	35.8	6.9	28.9	11.3	46.6	0.005

**00.00 – 02.59**	25.6	17.2	8.4	-11.0	27.8	0.409

**03.00 – 05.59**	10.2	34.5	24.3	-4.6	44.0	0.015

**06.00 – 08.59**	0.0	6.9	6.9	-16.1	2.3	0.178

**Weekday **	2.3	2.4	0.1	-1.2	1.1	0.996

**12.00 – 14.59**	13.3	15.2	1.8	-19.1	15.4	0.712

**15.00 – 17.59**	13.3	15.2	1.8	-19.1	15.4	0.712

**18.00 – 20.59**	3.3	0.0	3.3	-3.1	9.8	0.476

**21.00 – 23.59**	23.3	12.1	11.2	-7.6	30.0	0.242

**00.00 – 02.59**	33.3	24.2	9.1	-13.2	31.4	0.425

**03.00 – 05.59**	00.0	18.2	18.2	-31.3	-5.0	0.025

**06.00 – 08.59**	00.0	9.1	9.1	-18.9	0.7	0.239

There was a non-statistically significant reduction in the proportion of alcohol related attendances at the weekend in 2006 compared to 2005. In 2006, significantly more weekend alcohol related attendances occurred in the early hours of the morning than in 2005. Conversely, in 2005, significantly more alcohol related attendances occurred earlier, between 21.00–23.59 (Table 1).

The distribution of alcohol related attendances by time of day in the weekday was similar between 2005 and 2006, although there were no alcohol related attendances between the hours of 03.00 and 08.59 in 2005, compared to 27.3% in 2006 (difference 27.3%, 95% confidence interval 12.1% to 42.5%, p < 0.001).

## Discussion

There was no increase in the number of alcohol related attendances or subsequent alcohol related hospital admissions between our selected weeks in 2005 and 2006 – before and after the Act. We also noted no significant difference in the days of the week of alcohol related attendances between 2005 and 2006.

Interestingly, at the weekend in 2006, a significantly greater proportion of attendances were observed in the early hours of the morning (between 3 and 6 am), and a significantly smaller proportion in the earlier evening (between 6 pm and midnight). This trend in attendances occurring during the early hours of the morning was also seen for weekdays. Our findings suggest that although the Act has not affected the number of alcohol related attendances at the Emergency Department or the day of presentation; it may have shifted the time of attendances into the early hours of the morning.

Our findings support those of Graham *et al*, who found that restricting the availability of extensions to licensing hours led to no significant increases in alcohol related attendance, although studies overseas have shown that increased alcohol availability is causally associated with use and alcohol related illness and injury [15-17]. These studies encompassed several variables including the price of alcohol, opening hours and age-restrictions. There is also a consequential impact of alcohol availability on health services [18-20]. Reported rates of alcohol related attendances are as high as 35% [14]. This is in marked contrast to the lower rates found in our study, though our rates are similar to those in a comparable recent study by Newton *et al *[13]. Furthermore, their study period was limited to overnight cases, when alcohol related attendances are known to be higher. The variation in reported rates of alcohol related attendances is likely to reflect the variable definition of the term 'alcohol related attendance' [13]. We excluded patients with alcohol related medical problems who had no documentation of recent alcohol consumption. Staff at City Hospital in Birmingham do not routinely record inconsequential alcohol consumption in the notes, which may partly account for the low rates in our study. Furthermore, in Birmingham the proportion of the population from an ethnic group other than white is three times the national average [21]. All minority ethnic groups are recognised to consume less alcohol which may also account for the low rates in our study. Newton *et al *also recently found an increase in both alcohol related attendances and admissions since the Act, however, it only included attendances between 9 pm and 9 am. A shift in the time of attendance may explain the reported increase and difference with our study [13].

Retrospective studies relying on medical notes are notoriously incomplete. Levels of alcohol-related attendances may be under-estimated by our study due either to lack of reference to alcohol involvement in the notes or to patients not disclosing the involvement of alcohol. Other methods for detecting alcohol involvement include blood alcohol measurement or the use of screening questionnaires; a range of which are currently being trialed by the Screening and Intervention Programme for Sensible drinking projects (SIPS) [22]. We did not use a pro-forma in our study to avoid introducing bias to the post-Act cohort.

Our study period was limited to a week in each year, therefore it would be useful to conduct a study over a longer time period incorporating a larger cohort of patients to confirm our findings. The Act was implemented on the 24^th ^November 2005 and in the period since implementation, until the start of our study period in 2006, 37% (1036/2800) of licensed premises in Birmingham had successfully applied to extend their opening hours [23]. This is in keeping with our result of a temporal shift in alcohol related attendances.

Although two months had elapsed since the Act, some licensed premises may not have changed their opening hours immediately. Attitudes towards alcohol may have taken some time to adapt to the new environment, and the 'novelty value' of longer opening hours may wear off with a reduction in alcohol consumption. Although repeat studies at later dates may illicit further findings with regard to the Act's effects, the further the distance from the introduction of the Act, the less likely changes can be related solely to it.

Our study was carried out over a one-week period in January. There were no planned major sporting or musical events in the area surrounding the emergency department that were likely to have affected alcohol consumption. There are well-established seasonal variations in alcohol consumption [24]. Any potential bias was minimised by choosing the same week in each January. Other factors known to affect alcohol related attendances to the emergency department include the fluctuating cost of alcohol, and a reduction in overall spending in January, which may have impacted on our study weeks [25-28].

## Conclusion

Our study shows that there was a small change in the pattern of alcohol related attendances to the Emergency Department. Changes introduced by the Act may have resulted in a dissipation of the attendances related to alcohol, with a greater proportion of attendances occurring during working hours and in the early morning after 03:00. This may reflect a change in alcohol consumption patterns. This has implications to the NHS and additionally the Police, particularly with regard to night-time services and NHS staff requirements for the care of patients with alcohol related injuries and health problems.

## Competing interests

The authors declare that they have no competing interests.

## Authors' contributions

Authorship-All named authors have contributed substantially to the study design, data collection and data interpretation and the preparation of the manuscript. All named authors have read and approved the final version of the manuscript.

## Pre-publication history

The pre-publication history for this paper can be accessed here:


